# The Global Burden of Visual Difficulty in Low, Middle, and High Income Countries

**DOI:** 10.1371/journal.pone.0063315

**Published:** 2013-05-10

**Authors:** Ellen E. Freeman, Marie-Hélène Roy-Gagnon, Elodie Samson, Slim Haddad, Marie-Josée Aubin, Claudia Vela, Maria Victoria Zunzunegui

**Affiliations:** 1 Department of Ophthalmology, Université de Montréal, Montreal, Quebec, Canada; 2 Centre de Recherche, Hôpital Maisonneuve-Rosemont, Montreal, Quebec, Canada; 3 Centre de Recherche de Centre Hospitalier de l’Université de Montréal, Montreal, Quebec, Canada; Boston Children’s Hospital, United States of America

## Abstract

**Purpose:**

Using a world-wide, population-based dataset of adults, we sought to determine the frequency of far visual difficulty and its associated risk factors.

**Methods:**

The World Health Survey (WHS) was conducted in 70 countries throughout the world in 2003 using a random, multi-stage, stratified, cluster sampling design of adults ages 18 years and older. Far vision was assessed by asking “In the last 30 days, how much difficulty did you have in seeing and recognizing a person you know across the road (i.e. from a distance of about 20 meters)?”. Responses included none, mild, moderate, severe, or extreme/unable. The income status of countries was estimated using gross national income per capita data from 2003 from the World Bank. Prevalence and regression estimates were adjusted to account for the complex sample design.

**Results:**

21% of adults reported any visual difficulty. The rate varied by the income status of the country with the percentage who had any visual difficulty being 24%, 23%, and 13% in low, middle, and high income countries, respectively. Five percent of people reported severe or extreme visual difficulty with rates in low, middle, and high income countries of 6%, 5%, and 2% respectively. Risk factors for visual difficulty included older age, female sex, poorer socioeconomic status, little to no formal education, and diabetes (P<0.05).

**Conclusions:**

One out of five adults in the WHS reported some degree of far visual difficulty. Given the importance of vision to living an independent life, better access to quality eye care services and life course factors affecting vision health (e.g. repeated eye infections, diet lacking vitamin A) must receive adequate attention and resources, especially in low and middle income countries.

## Introduction

The most recent world-wide estimates of visual impairment and blindness are 285 million and 39 million, respectively [1]. These estimates are based on 53 population-based surveys that measured presenting far visual acuity from 39 countries of the world. These estimates were then imputed to countries without data to give a global estimate. Much of the data from these surveys were from rapid assessments for cataract surgery services and were limited to adults ages 50 years and older. Estimates for some world regions were based on a very small number of countries. Furthermore, no attempts were made to measure visual difficulty. Researchers have increasingly recognized the importance of asking about visual difficulty or vision-related quality of life as visual acuity may not accurately reflect all aspects of visual disability [2], [3], [4], [5].

The World Health Survey (WHS), which was carried out in adults in 2002–2003 in 70 countries of the world [6], provides another source of data to understand the global burden of vision loss. The WHS does not have data on visual acuity but rather asked about the level of difficulty that participants had seeing and recognizing a person across the road (i.e. about 20 meters). Our goals for this analysis were to use the WHS data to 1) provide country-specific prevalence estimates for far visual difficulty, 2) create a map highlighting the countries in the world with the most far visual difficulty, 3) identify risk factors for far visual difficulty and explore whether they differ by the income status of the country.

## Methods

### World Health Survey

#### Ethics Statement

Informed consent was obtained from all participants and ethics approval was obtained by local institutional review committees. In addition, the ethics committee of Hôpital Maisonneuve-Rosemont, where the analyses were conducted, approved the project. Numerous papers have now been published using this global dataset [7], [8], [9], [10], [11].

#### Study population

The goal of the WHS, which was coordinated by the World Health Organization (WHO), was to collect population-based, nationally representative, cross-sectional data from 70 countries within 6 world regions. Data were collected from 276,647 people from 30 European countries, 18 African countries, 7 North and South American, 4 Eastern Mediterranean, 5 Southeast Asian, and 6 Western Pacific countries in 2002–2003. Survey institutions were selected by WHO in each country and these institutions carried out the survey according to WHS procedures.

#### Sampling strategy

A multi-stage stratified random cluster sampling strategy was used to identify the participants to be contacted in each country. Strata were created based on 3 factors: region, socioeconomic status, and presence of a healthcare facility. Lists of households were obtained from population registries, voter lists, manual enumeration, or other methods. Households within the sampling units were randomly selected from these lists. Within each household, an adult 18 years or older was randomly selected using a Kish table to complete the survey. If the selected member of the household was in an institution, the survey team travelled to the institution. Non-response was carefully documented.

#### Survey administration

All surveys were interviewer-administered in person in local languages. Questionnaires were translated into 68 local languages using standard techniques. Briefly, forward translation was done locally by a bilingual multidisciplinary group. Back-translation was then done by an independent group. A review of the back-translation was also done at WHO. Any discrepancies were resolved. A review of the translated instrument was then done by a panel of experts.

#### Vision data and its validity

Far vision was assessed with the following question: “In the last 30 days, how much difficulty did you have in seeing and recognizing a person you know across the road (i.e. from a distance of about 20 meters)?”. Possible responses included none, mild, moderate, severe, and extreme/unable. The validity of a self-report question similar to this was demonstrated in Klein *et al* who found a moderate correlation between positive answers to questions about vision and visual acuity loss measured with a visual acuity chart [12].

Furthermore, we examined the validity of the exact WHS question in a sample of 139 patients recruited from an ophthalmology clinic in Montreal, Canada. The logarithm of the minimum angle of resolution (logMAR) visual acuity of these patients ranged from 0.0 (i.e. normal or 20/20) to 1.5 (i.e. blind or 20/632). Responses to the question were moderately correlated with logMAR visual acuity (Pearson’s r = 0.57, P<0.05). The mean logMAR scores of those who reported none, mild, moderate, severe, and extreme visual difficulty were 0.12, 0.30, 0.36, 0,52, and 0.60. Thus, a response of mild visual difficulty or worse may have closely corresponded to North American definitions of visual impairment (20/40 or 0.30 logMAR), while a response of severe visual difficulty may have corresponded to WHO definitions of visual impairment (20/60 or 0.48 logMAR).

#### Other relevant data collection

Demographic information was obtained on age, gender, and highest level of formal education completed. The wealth of the participant was estimated by measuring asset ownership using the method of Filmer and Pritchett [13]. The wealth of each participant was assessed by asking 15–20 questions on asset ownership. The assets included in the questions for low and middle income countries (i.e. bucket, electricity, refrigerator) were not exactly the same as those used for high income countries (i.e. car, television set, computer).

A principal components analysis (PCA) was then done separately for each country by us to determine the weights to create an index of the asset variables. The weights for the first component were then applied to each person’s data giving a continuous asset index measure. We then categorized this index into high, medium, and low tertiles. This asset index had good properties in an Indian dataset with good internal coherence and robustness to the selection of variables used [13]. The index reflects the long-term economic position of a household rather than its current economic status and gives the position of a person within his or her country. Because the PCA was done separately for each country, the absolute value of the wealth index cannot be compared between countries.

Also, in low and middle income countries only, questions were asked about fruit and vegetable consumption per day and participants were asked about a physician diagnosis of diabetes.

### Income Status of Country

We added data to the WHS on the income status of the 70 countries using data from 2003 from the World Bank website [14]. According to the website, gross national income was converted to US dollars using the Atlas method and was divided by the mid-year population. The Atlas method of conversion is used by the World Bank to smooth fluctuations in prices and exchange rates. We categorized this variable into 3 categories using World Bank classifications. The 3 categories were low (<$766), middle ($766–$9385), and high income (>$9385) [14].

### Data Compilation and Cleaning

We downloaded the WHS datasets for each country from the WHS website (http://www.who.int/healthinfo/survey/en/index.html) and appended them together to create a single dataset. We checked and cleaned the data to eliminate implausible values, ineligible persons, and to exclude certain people without sufficient data. For example, 9,571 people were excluded who did not answer any questions in the individual questionnaire and 367 people were excluded who were listed as less than 18 years old. Our final dataset for analysis contained 276,647 people.

### Data Analysis

260,958 people (94%) answered the question on far vision and are the focus of this analysis. Means, standard errors, and percentages were estimated and were adjusted for the complex survey design. Eleven countries (Austria, Belgium, Germany, Denmark, United Kingdom, Greece, Italy, Netherlands, Slovenia, Guatamala, Zambia) did not report survey design information and therefore unweighted estimates are given for these 11 countries. Prevalence estimates were adjusted for age using direct adjustment. The WHO World Standard population was used [15].

Differences in means and proportions were tested using ANOVA and Pearson’s chi square tests taking into account the complex survey design. Logistic regression was performed to identify factors associated with far visual difficulty while adjusting for other factors. The factors that we examined included demographic factors like age, gender, education, and wealth, disease factors like diabetes, and dietary factors like fruit and vegetable consumption. We chose these factors based on prior literature that indicated possible associations with vision loss [16], 17,18,19. Interactions between each risk factor and the income status of the country were examined by stratification and were tested by adding interaction terms to the regression model. Regression analyses took into account the complex survey design and therefore excluded the 11 countries noted above that lacked data on survey design. Analyses that accounted for the complex survey design were done using the variables for sampling weight, strata, and primary sampling unit. Then, the survey estimation (SVY) commands in STATA/IC software version 11.2 were used (StataCorp, College Station Texas, USA) with standard errors corrected using Taylor linearized variance estimation. The map was done in SAS Version 9.2 (SAS Corp, Cary, NC, USA).

## Results

**Figure 1:**
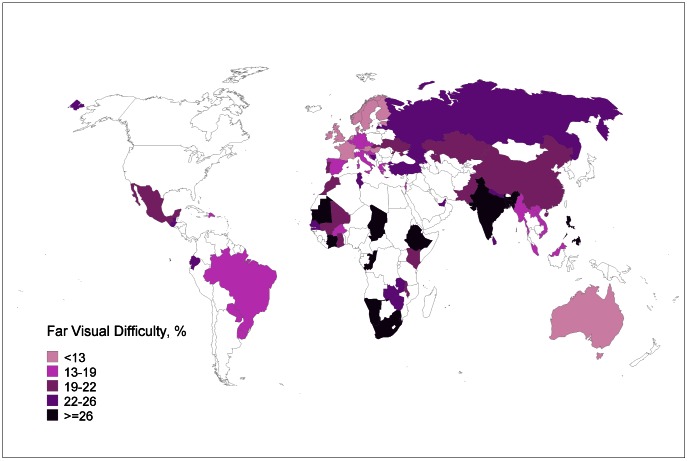
Map showing the degree of any difficulty with far vision. Darker colors indicate a greater degree of any degree of far visual difficulty. Countries in white did not participate in the World Health Survey.

The mean age of the sample was 39.1 years old (95% CI 38.9, 39.3). The global prevalence of any far visual difficulty was 21%. In adults less than 50 years old, the prevalence of any far visual difficulty was 13%, while in adults 50 years or older, the prevalence was 36%. For low, middle, and high income countries, the age-adjusted prevalence rate of any far visual difficulty was 24%, 23%, and 13%, respectively. In Table 1, the age-adjusted prevalence rates are given by country. The lowest rates of any far visual difficulty were found in Norway, Australia, and Sweden, while the highest rates were found in Comoros, Mauritania, and the Philippines. The data are summarized on a map in Figure 1.

**Table 1:** Age-adjusted prevalence of any visual difficulty and severe or extreme visual difficulty by region and country*.

Region	Country (Income Status)	Any Far Visual Difficulty Prevalence	95% CI	Severe or Extreme Far Visual Difficulty Prevalence	95% CI
Africa	Burkina Faso (LIC)	19.0%	16.9, 21.1%	5.5%	4.3, 6.8%
	Chad (LIC)	34.5%	32.0, 37.1%	6.7%	5.7, 7.8%
	Côte d’Ivoire (LIC)	27.9%	25.6, 30.3%	6.5%	5.1, 7.9%
	Congo (LIC)	31.6%	24.4, 38.9%	4.8%	2.3, 7.2%
	Comoros (LIC)	49.4%	46.2, 52.7%	9.3%	7.8, 10.9%
	Ethiopia (LIC)	26.0%	24.3, 27.7%	6.0%	5.0, 7.0%
	Ghana (LIC)	20.3%	18.8, 21.9%	5.2%	4.4, 6.0%
	Kenya (LIC)	20.9%	18.3, 23.6%	4.7%	3.8, 5.6%
	Mali (LIC)	19.6%	16.1, 23.0%	4.0%	2.7, 5.4%
	Mauritania (LIC)	42.6%	39.4, 45.9%	9.4%	7.2, 11.6%
	Malawi (LIC)	19.1%	17.0, 21.3%	6.4%	5.5, 7.4%
	Mauritius (MIC)	24.0%	21.6, 26.4%	6.5%	5.4, 7.5%
	Namibia (MIC)	32.8%	30.1, 35.4%	7.3%	5.9, 8.7%
	Senegal (LIC)	24.3%	21.9, 26.7%	7.1%	5.5, 8.7%
	Swaziland (MIC)	35.2%	33.1, 37.2%	15.4%	13.0, 17.7%
	South Africa (MIC)	32.7%	29.0, 36.4%	7.9%	5.6, 10.2%
	Zambia (LIC)^†^	25.7%	24.2, 27.2%	5.1%	4.2, 5.9%
	Zimbabwe (LIC)	23.1%	21.1, 25.1%	7.5%	6.3, 8.8%
Americas	Brazil (MIC)	18.9%	17.5, 20.3%	6.0%	5.1, 6.9%
	Dominican Republic(MIC)	18.6%	16.9, 20.3%	5.7%	4.7, 6.8%
	Ecuador (MIC)	23.7%	22.0, 25.4%	5.9%	4.8, 6.9%
	Guatamala (MIC)^†^	25.6%	24.4, 26.8%	7.8%	7.1, 8.6%
	Mexico (MIC)	21.9%	21.1, 22.6%	3.7%	3.4, 4.0%
	Paraguay (MIC)	16.8%	15.7, 17.9%	5.0%	4.3, 5.8%
	Uruguay (MIC)	13.1%	10.3, 15.9%	2.1%	1.8, 2.5%
Europe	Austria (HIC)^†^	12.7%	10.5, 14.8%	0.6%	0.2, 1.1%
	Belgium (HIC)^†^	13.7%	11.3, 16.1%	1.7%	1.0, 2.5%
	Bosnia and Herzegovina (MIC)	25.1%	20.5, 29.6%	3.8%	2.1, 5.6%
	Croatia (MIC)	13.5%	10.9, 16.1%	2.7%	1.8, 3.6%
	Czech Republic (MIC)	17.2%	12.5, 21.8%	1.3%	0.7, 2.0%
	Denmark (HIC)^†^	7.3%	5.3, 9.4%	1.3%	0.6, 1.9%
	Estonia (MIC)	12.3%	9.7, 14.8%	2.6%	1.4, 4.0%
	Finland (HIC)	10.2%	7.6, 12.8%	0.5%	0.1, 0.9%
	France (HIC)	11.1%	7.9, 14.4%	0.9%	0.4, 1.4%
	Georgia (MIC)	22.6%	19.6, 25.5%	4.6%	3.6, 5.5%
	Germany (HIC)^†^	15.1%	12.7, 17.4%	2.1%	1.1, 3.0%
	Greece (HIC)^†^	15.5%	11.7, 19.3%	2.0%	1.1, 2.9%
	Hungary (MIC)	9.3%	8.0, 10.7%	2.8%	2.1, 3.5%
	Ireland (HIC)	10.1%	7.8, 12.5%	0.8%	0.2, 1.4%
	Israel (HIC)	15.2%	12.6,17.9%	3.4%	2.2, 4.6%
	Italy(HIC)^†^	15.5%	13.3, 17.7%	1.6%	0.9, 2.3%
	Kazakhstan (MIC)	21.7%	18.7, 24.7%	3.8%	2.5, 5.2%
	Latvia (MIC)	24.6%	20.4, 28.8%	3.7%	2.5, 5.0%
	Luxembourg (HIC)	9.5%	7.2, 11.7%	1.0%	0.3, 1.6%
	Netherlands (HIC)^†^	7.8%	6.1, 9.5%	1.0%	0.4, 1.7%
	Norway (HIC)	5.7%	4.1, 7.3%	1.0%	0.4, 1.6%
	Portugal (HIC)	19.2%	15.9, 22.6%	2.9%	2.0, 3.8%
	Russian Federation (MIC)	25.2%	21.7, 28.6%	4.2%	3.4, 5.0%
	Slovakia (MIC)	20.6%	16.6, 24.5%	2.4%	0.1, 4.9%
	Slovenia (HIC)^†^	17.3%	14.1, 20.5%	3.2%	1.9, 4.5%
	Spain (HIC)	15.0%	13.7, 16.3%	2.2%	1.8, 2.6%
	Sweden (HIC)	8.1%	5.3, 10.9%	2.5%	0.8, 4.1%
	Turkey (MIC)	23.6%	22.3, 24.9%	6.6%	5.9, 7.4%
	Ukraine (MIC)	19.1%	17.3, 21.0%	4.3%	3.3, 5.3%
	United Kingdom (HIC)^†^	9.2%	7.3, 11.0%	1.6%	0.9, 2.3%
Eastern Mediterranean	Morocco (MIC)	19.9%	17.7, 22.1%	10.1%	8.3, 11.9%
	Pakistan (LIC)	21.4%	19.8, 23.0%	2.9%	2.2, 3.6%
	Tunisia (MIC)	22.5%	20.8, 24.1%	6.0%	5.2, 6.8%
	United Arab Emirates (HIC)	25.5%	21.2, 29.9%	3.6%	2.0, 5.2%
Southeast Asia	Bangladesh (LIC)	28.5%	27.1, 29.8%	10.7%	9.8, 11.7%
	India (LIC)	27.0%	24.8, 29.1%	8.6%	7.3, 9.9%
	Myanmar (LIC)	18.6%	16.8, 20.4%	2.9%	2.3, 3.5%
	Nepal (LIC)	25.2%	24.1, 26.3%	9.5%	8.8, 10.2%
	Sri Lanka (MIC)	22.2%	20.2, 24.2%	3.6%	2.8, 4.4%
Western Pacific	Australia (HIC)	7.1%	5.5, 8.7%	0.6%	0.3, 0.9%
	China (MIC)	20.4%	15.8, 24.9%	1.5%	1.1, 2.0%
	Lao People’s Democratic Republic (LIC)	17.5%	16.1, 18.8%	3.1%	2.4, 3.7%
	Malaysia (MIC)	15.6%	14.3, 16.9%	1.6%	1.2, 2.1%
	Philippines (MIC)	38.9%	36.8, 40.9%	6.6%	5.8, 7.4%
	Vietnam (LIC)	17.1%	14.2, 19.9%	3.0%	1.3, 4.7%

* Prevalence estimates and standard errors are adjusted for the complex survey design except for 11 countries (Austria, Belgium, Germany, Denmark, United Kingdom, Greece, Italy, Netherlands, Slovenia, Guatamala, Zambia) that did not provide information on sampling weights. Unweighted estimates are used for those 11 countries marked with †.

LIC = low income status, MIC = middle income status, HIC = high income status.

Five percent of the WHS sample reported severe or extreme visual difficulty. The age-adjusted prevalence rate of severe or extreme visual difficulty in low, middle, and high income countries was 6%, 5%, and 2%, respectively. The lowest rates of severe or extreme far visual difficulty were found in Finland, Australia, and Austria, while the highest rates were found in Swaziland, Bangladesh, and Morocco.

In Table 2, descriptive statistics are provided for WHS participants stratified by the income status of the country. The mean age of participants in low income countries (LIC) was 36.8, while it was 40.5 in middle-income countries (MIC) and 46.5 in high-income countries (HIC) (P<0.05). A large percentage (41%) of participants in LIC had no formal education compared to 7% and 2% of those in MIC and HIC (P<0.05). Fruit consumption was higher in MIC than in LIC while vegetable consumption was higher in LIC than in MIC (P<0.05). More people in MIC were diagnosed with diabetes than in LIC (P<0.05).

**Table 2:** Description of participants by income status of country*.

	Low Income Countries (20 countries, n = 90,158)	Middle Income Countries (28 countries, n = 145,342)	High Income Countries (11 countries, n = 25,458)
Risk Factor	% or mean (SE)	% or mean (SE)	% or mean (SE)
Age	36.8 (0.1)	40.5 (0.1)	46.5 (0.5)
Female gender	49%	52%	51%
Education Completed			
> = Secondary School	27%	65%	76%
Primary School	19%	19%	16%
<Primary School	13%	9%	6%
No Formal Education	41%	7%	2%
Wealth Index			
Low	40%	36%	35%
Middle	41%	41%	41%
High	20%	23%	24%
Fruit Consumption/Day			
0 servings	28%	15%	
1	35%	39%	
> = 2	38%	46%	
Vegetable Consumption/Day			
0 servings	4%	6%	
1	31%	42%	
> = 2	65%	53%	
Diagnosed with Diabetes	2%	5%	

* Statistically significant differences across income strata were found for all risk factors (p<0.05). SE = standard error.

In Table 3, risk factors for any far visual difficulty were examined. Older age was a risk factor for far visual difficulty in countries of all income strata (P<0.01) with odds ratios ranging from 1.03 (95% CI 1.02, 1.03) in HIC to 1.07 (95% CI 1.07, 1.08) in LIC. Female gender was a risk factor only in LIC (OR = 1.74, 95% CI 1.59, 1.89) and MIC (OR = 1.37, 95% CI 1.27, 1.47) (P<0.01). Lower education was a risk factor in all income strata (P<0.01). Having no formal education was a stronger risk factor in HIC (OR = 3.45, 95% CI 2.29, 5.22) than in LIC (OR = 1.33, 95% CI 1.16, 1.53) (interaction term P = 0.03). Similarly, a high wealth index within one’s country was protective in all income strata but it was more strongly protective in MIC (OR = 0.69, 95% CI 0.61, 0.78) (interaction term P<0.01) and HIC (OR = 0.54, 95% CI 0.41, 0.70) (interaction term P = 0.055). Eating two or more servings of fruit per day was protective for far visual difficulty in MIC (OR = 0.86, 95% CI 0.77, 0.98) while it was not in LIC. Eating 1 vegetable serving per day was protective in LIC (OR = 0.76, 95% CI 0.62, 0.93) but eating 2 or more servings was not protective. There was no association between vegetable consumption and far visual difficulty in MIC. A diagnosis of diabetes was a risk factor for far visual difficulty in both LIC (OR = 1.52, 95% CI 1.18, 1.97) and MIC (OR = 1.37, 95% CI 1.17, 1.60) but the association was stronger in LIC (interaction term P<0.01).

**Table 3:** Risk factors for any far visual difficulty by income status of country via multiple logistic regression.

	Low Income Countries95% CI		Middle Income Countries		High Income Countries	
Risk Factor	OR		OR	95% CI	OR	95% CI
Age	1.07	1.07, 1.08	1.05	1.04, 1.05	1.03	1.02, 1.03
Female gender	1.74	1.59, 1.89	1.37	1.27, 1.47	1.22	0.97, 1.53
Education Completed						
> = Secondary School	1.00		1.00		1.00	
Primary School	1.04	0.91, 1.19	1.10	1.00, 1.21	1.76	1.34, 2.32
<Primary School	1.26	1.09, 1.46	1.39	1.21, 1.59	2.66	1.82, 3.90
No Formal Education	1.33	1.16, 1.53	1.06	0.93, 1.21	3.45	2.29, 5.22
Wealth Index						
Low	1.00		1.00		1.00	
Middle	0.84	0.76, 0.92	0.82	0.74, 0.90	0.67	0.53, 0.86
High	0.83	0.72, 0.95	0.69	0.61, 0.78	0.54	0.41, 0.70
Fruit Consumption/Day						
0 Servings	1.00		1.00			
1	0.99	0.88, 1.12	0.98	0.87, 1.09		
> = 2	1.00	0.90, 1.12	0.86	0.77, 0.98		
Vegetable Consumption/Day						
0 Servings	1.00		1.00			
1	0.76	0.62, 0.93	1.07	0.88, 1.29		
> = 2	0.84	0.68, 1.04	0.90	0.74, 1.08		
Diabetes	1.52	1.18, 1.97	1.37	1.17, 1.60		

In sensitivity analyses that do not account for the complex survey design, we examined the impact of the absence of the 11 countries on our estimates of visual difficulty in LIC, MIC, and HIC. Including the 11 countries, the percentages with far visual difficulty were 25%, 23%, and 13%, respectively. Not including the 11 countries, the percentages were 25%, 23%, 14%. For severe visual difficulty, the percentages were 6.3%, 4.9%, and 1.9% with the 11 countries, while without the 11 countries, the percentages were 6.4%, 4.8%, and 2.0%. Because these percentages are quite similar to each other, this leads us to conclude that the absence of the 11 countries does not significantly bias our global estimates.

## Discussion

Twenty-one percent of the WHS sample reported some degree of far visual difficulty while 4.8% reported severe or extreme difficulty. In general, the percentage with far visual difficulty increased as the income level of the country decreased. However, there were some MIC with very high age-adjusted rates of any far visual difficulty such as the Philippines, Swaziland, and South Africa, which all had age-adjusted prevalence rates over 30%. Also, Morocco (MIC) had the third highest rate of severe or extreme visual difficulty. Comoros and Mauritania, two LIC, had the highest age-adjusted rates of any far visual difficulty with both over 40%. Swaziland (MIC) and Bangladesh (LIC) had the highest age-adjusted rates of severe or extreme far visual difficulty at 15.4% and 10.7%. The region with the largest burden was Africa followed by Southeast Asia.

We compared some of the highest rates of visual difficulty in the WHS to what can be found in prior published research. A rapid assessment of avoidable blindness was done in the Philippines in 2007 in adults ages 50 years and over [20]. Vision was measured using a tumbling E visual acuity chart. Using a cutoff of 6/18 (20/60 or logMAR 0.48) to define visual impairment, the authors found that 11% had visual impairment in Negros Island and 7.3% had visual impairment in the Antique district [20]. In our analysis using WHS data from the Philippines, 10.9% of people ages 50 years and older reported severe or extreme visual difficulty, which may correspond to a cutoff of 20/60 according to our validation study. These results are very similar. By contrast, a population-based study done in Cape Town, South Africa reported a prevalence of visual impairment of 4.9% [16] using the same cutoff in people ages 50 years and older while our estimate for adults ages 50 and older for South Africa is much higher at 13.8%. This may indicate that visual impairment is a much bigger problem in the rural regions of South Africa, which were included in the WHS, than in an urban location like Cape Town. We did not find any literature describing the prevalence of visual impairment in the rural regions of South Africa. Furthermore, we did not find any published prevalence studies for Swaziland, Comoros, or Mauritania.

Demographic risk factors for visual difficulty varied by the income status of the country. For example, female gender was a risk factor for visual difficulty in LIC and MIC but not in HIC. This may be because women in LIC and MIC have not attained the status that women have attained in HIC according to data on gender inequality from the United Nations [21]. They have less financial resources and empowerment, which are necessary to access eye care and to lead a healthy life, than women in HIC. By contrast, low education was a risk factor for far visual difficulty in countries of all income strata but it was a stronger risk factor in HIC. This could be explained by the fact that education is less helpful if one does not have access to eye care services as might be the case in many LIC and MIC. In HIC, access to care is relatively less of a problem but one has to be educated in order to know that a vision problem may be treatable and how to use the eye care services that exist. Higher education is known to be an important factor in explaining eye care utilization [22], [23], [24]. One also needs to be able to afford and travel to the services that exist. The wealth index was associated with far visual difficulty in countries of all income strata although the association was strongest in HIC.

Our results on fruit and vegetable consumption were inconsistent. Fruit and vegetables contain antioxidants and vitamins that may protect against cataract [25] and age-related macular degeneration [26], and do protect against xerophthalmia (vitamin A deficiency) [27]. We found consumption of two or more servings of fruit per day was associated with less far visual difficulty but only in MIC and not in LIC. We did not have data on fruit consumption in HIC. One would expect to find this relationship in both LIC and MIC if one truly existed, and therefore, it is possible that this is a chance finding or that the WHS questions on fruit and vegetable consumption were not detailed enough. Little prior research has been done on fruit consumption and vision loss/eye disease in low and middle income countries [28], [29]. Our findings were also inconsistent for vegetable consumption.

This is the first study to examine visual difficulty in such a large number of countries using the same data collection protocol. The WHS was done in all adults ages 18 years and older as opposed to other population-based studies which have largely focused on adults ages 50 years and older. These data provide complementary information to other studies on visual impairment and can be used to provide information on countries which have no previously published data on visual impairment. Furthermore, we added data from the World Bank on the income status of the country to investigate interactions.

A limitation of this study is that visual acuity was not measured and causes of vision loss were not investigated. Although visual difficulty is a valuable outcome by itself, using it as a proxy for visual acuity may be problematic. Some respondents may have over or under-estimated their visual difficulty due to cultural or other factors. For this reason, in Montreal, Canada, we examined the validity of the vision question used in the WHS and found moderate correlation with the ETDRS distance visual acuity chart. Furthermore, the cutoffs of mild and severe visual difficulty corresponded well with acuity levels of 20/40 and 20/60 respectively. Whether this question correlates well with visual acuity in countries with less formal education and different cultures compared to Montreal is unknown. Another limitation of this study is its cross-sectional nature which precludes establishing temporality of the risk factors and the onset of visual difficulty. Finally, the data we present here are 10 years old. It is possible that interventions and increased funding directed towards eye disease during that time period have changed the degree of visual difficulty in certain countries. For example, the prevalence of trachoma trichiasis has decreased in Ethiopia after widespread implementation of the SAFE strategy (surgery, antibiotics, face washing, environmental hygiene) [30]. Vitamin A deficiency has been on the decline in many areas due to widespread supplementation [31]. Onchocerciasis has been targeted in central and eastern Africa with mass treatment of ivermectin [32]. However, despite the age of the WHS data, our results may still have tremendous value to indicate the need for attention in certain areas that have been neglected.

In conclusion, one out of five adults in the WHS reported any far visual difficulty while one out of twenty reported severe or extreme visual difficulty. Those with low levels of formal education and women in low and middle income countries were most affected. The leading causes of blindness in the world are known to be cataract and refractive error: two very treatable conditions [33], [34]. Other important causes of blindness listed in order of prevalence are glaucoma, age-related macular degeneration, corneal opacities, diabetic retinopathy, childhood blindness, trachoma, and onchocerciasis [34]. Great progress has been made for some of these conditions [30], [31], [32]. Given the importance of vision to living an independent life, quality eye care service delivery and life course factors affecting vision health (e.g. repeated eye infections, diet lacking vitamin A) must receive adequate attention and resources, especially in low and middle income countries.
